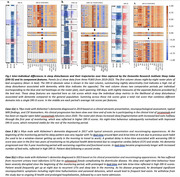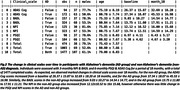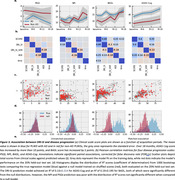# Digital biomarkers of sleep and nocturnal behaviour derived from contactless longitudinal sleep monitoring predict cognitive decline and deterioration in daily function in people living with dementia

**DOI:** 10.1002/alz.091826

**Published:** 2025-01-09

**Authors:** Magdalena A. Kolanko, Eyal Soreq, Mara Golemme, Chloe Walsh, Martina Del Giovane, David J Sharp

**Affiliations:** ^1^ UK Dementia Research Institute Centre for Care Research and Technology, London UK; ^2^ Imperial College London, Department of Brain Sciences, London UK; ^3^ UK Dementia Research Institute Centre for Care Research and Technology, London UK; ^4^ UK Dementia Research Institute, Care Research and Technology Centre, London UK; ^5^ Surrey and Borders Partnership NHS Foundation Trust, Guildford UK

## Abstract

**Background:**

Growing evidence suggests sleep disturbance may promote Alzheimer’s disease (AD) pathology. However, the relationship between sleep and AD progression remains poorly defined due to lack of longitudinal sleep monitoring methods. We previously presented a novel approach for contactless sleep monitoring in people living with dementia (PLWD)^1^. Here, we examine the association between the Dementia Research Institute Sleep Index (DRI‐SI) ‐ an interpretable measure of sleep disturbances derived from contactless sensor data ‐ and clinical disease progression.

**Method:**

Withing’s Sleep Analyser was used to monitor 120PLWD taking part in a home‐monitoring study between 2019‐2023 (46k total nights). Participants were assessed 6‐monthly with ADAS‐Cog and Pittsburgh Sleep Quality Index (PSQI), and 3‐monthly with Neuropsychiatric Inventory (NPI) and Bristol Activities of Daily Living Scale (BADL). We used mixed linear models to examine the association between each assessment and the DRI‐SI score and variability. Additionally, a predictive model using an explainable boosting regressor was constructed to assess the DRI‐SI’s ability to predict ADAS‐Cog, PSQI, NPI and BADL scores.

**Result:**

We included 107 participants (69 AD, 38 non‐AD dementia) with sufficient contemporaneous questionnaire and sleep‐mat data. Minute‐to‐minute timeseries were extracted from the sleep‐mats to calculate nightly DRI‐SI scores (Figure 1). Longitudinally, there was a significant gradual decline in ADAS‐Cog and BADL scores over the 18‐month follow‐up, while the NPI and PSQI showed little change (Figure 2). The DRI‐SI score and variability were significantly associated with BADL (Coef.=2.0±0.6, Z=3.3, P=0.001; Coef.=3.9±1.7, Z=2.3, P=0.02). ADAS‐Cog and NPI were also significantly associated with the DRI‐SI score, but not the DRI‐SI variability (Coef.=3.4±1.1, Z=2.95, P=0.003; Coef.=3.1±0.9, Z=3.3, P=0.001). Interestingly, no association was seen between the DRI‐SI and PSQI. The predictive model demonstrated significant predictive power for ADAS‐Cog and BADL scores, indicating the potential of DRI‐SI as a digital biomarker for cognitive and daily function in dementia (Figure 3c,d).

**Conclusion:**

Our results suggest that DRI‐SI, a novel digital biomarker of sleep disturbance and night‐time behaviour in dementia derived from contactless remote sleep‐monitoring, associates with measures of cognition and function in dementia. As such, DR‐SI shows great promise for better predicting dementia progression, personalizing treatments, and augmenting outcomes in clinical trials.